# Live-cell imaging of chromatin contacts opens a new window into chromatin dynamics

**DOI:** 10.1186/s13072-023-00503-9

**Published:** 2023-06-23

**Authors:** Jente van Staalduinen, Thomas van Staveren, Frank Grosveld, Kerstin S. Wendt

**Affiliations:** Department of Cell Biology, Erasmus MC, Dr. Molewaterplein 50, 3015 GE Rotterdam, The Netherlands

## Abstract

Our understanding of the organization of the chromatin fiber within the cell nucleus has made great progress in the last few years. High-resolution techniques based on next-generation sequencing as well as optical imaging that can investigate chromatin conformations down to the single cell level have revealed that chromatin structure is highly heterogeneous at the level of the individual allele. While TAD boundaries and enhancer–promoter pairs emerge as hotspots of 3D proximity, the spatiotemporal dynamics of these different types of chromatin contacts remain largely unexplored. Investigation of chromatin contacts in live single cells is necessary to close this knowledge gap and further enhance the current models of 3D genome organization and enhancer–promoter communication. In this review, we first discuss the potential of single locus labeling to study architectural and enhancer–promoter contacts and provide an overview of the available single locus labeling techniques such as FROS, TALE, CRISPR–dCas9 and ANCHOR, and discuss the latest developments and applications of these systems.

## Introduction

Human diploid cells need to handle a genome consisting of little more than 6 Gigabases in a way that protects it against damage and can be propagated unchanged over generations. Genes need to be activated and silenced depending on external signals, cell-fate decisions and to maintain cell identity. This depends on a tightly controlled access of gene-regulatory proteins to the genome. At the same time, the whole genome undergoes major reorganization in proliferating cells. The entire functional genome is duplicated during replication and then highly compacted upon entry into mitosis to allow segregation of sister chromatids and equal distribution of the genome onto the daughter cells.

The packing of the DNA with its bound proteins (chromatin fiber) within the cell nucleus is fundamental for these processes. In interphase, the chromatin of individual chromosomes locates in distinct chromosome territories with little intermingling. Genome-wide conformation capture studies such as Hi-C revealed that chromosomes can be divided into more transcriptionally active regions (A compartment) and inactive regions (B compartment). These are marked by specific histone modifications and interact with the same type of regions while avoiding the other compartment. The driving forces between this compartmentalization are complex and involve the folding of the chromatin fiber, general transcriptional activity and clustering of proteins and DNA, invoking mechanisms of phase separation. Liquid–liquid phase separation (LLPS) is a little understood multifactorial process underlying the self-organization of membraneless chromatin compartments, driven by intrinsic properties of the chromatin fiber, associated RNA and chromatin-binding proteins via disordered domains (e.g., BRD4) and/or by accumulation of transcription activators (transcription factories, RNA Pol II) (for a review see [1]).

**Fig. 1:**
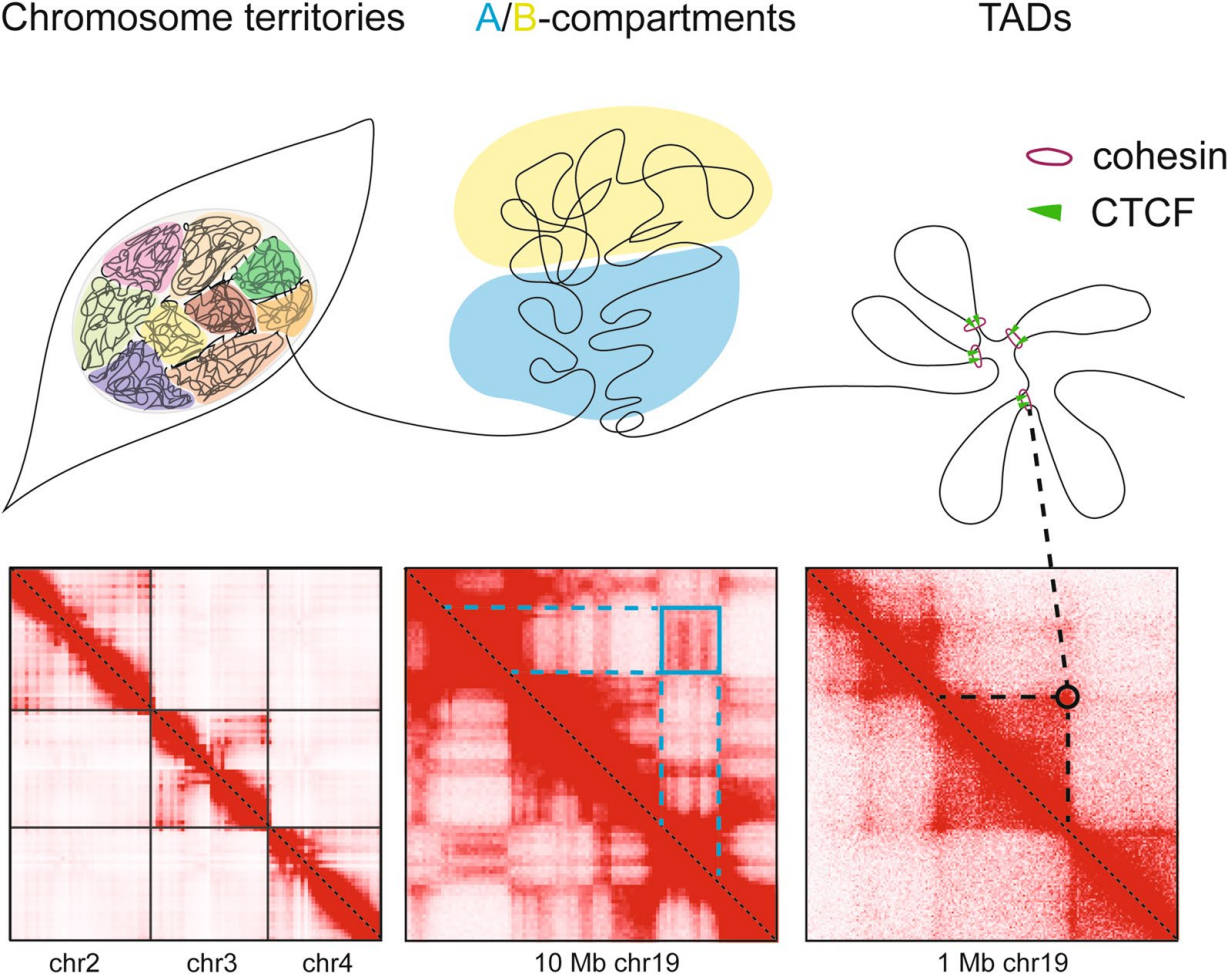
Organizational layers of chromatin and their reflection in Hi-C maps. Organizational layers of the chromatin fiber in the cell nucleus (top) aligned with the representation of those features in Hi-C interaction maps (bottom). Chromosomes reside in distinct territories with little intermingling. Therefore, chromatin contacts occur predominantly within the same chromosome (left panels). Within the chromosomes compartments with different chromatin properties (A compartment—active chromatin, B compartment—inactive chromatin) can be observed that interact with regions belonging to the same compartment type (blue box) (middle panels). Underlying these features in the folding of the chromatin fiber into chromatin loops, to a large extend mediated by the cohesin complex and CTCF, visible in the 1 Mb region shown for chr19 as regions with strong interactions (right panels). (Hi-C maps were generated using data for HCT116 cells by Rao et al. 2017 [5] and Juicebox.)

Chromatin conformation capture approaches revealed regions with strong directionality of chromatin contacts within these compartments, visible as characteristic triangles in contact maps and referred to as topologically associating domains (TADs) (Fig. 1). Contact maps obtained with higher resolution show that TADs consist of single or multiple contacts formed by distinct chromatin loops [2], which we refer to as “structural loops”, because they are very similar in different cell types. Methods like Hi-C generally process millions of cells and hence contact maps represent an average of a large cell population and do not yield information about the presence of the individual loops in individual cells.

Key proteins for the formation of structural loops are the cohesin complex and the chromatin insulator protein CTCF. Depletion of critical cohesin complex subunits such as RAD21 or CTCF leads to the apparent loss of chromatin loops [3–5]. Models for the establishment of chromatin loops by cohesin and CTCF suggest an intriguing mechanism of ATP-driven loop extrusion by the cohesin complex that is halted when the extrusion complex encounters two convergently oriented CTCF sites [6, 7]. Within these structural loops more loops are formed between enhancers and promoters which we refer to as “functional loops”. Among the players involved in the formation and positioning of such loops are RNA polymerase II [8], the Mediator complex [9] transcription factors and cohesin. A reorganization of chromatin loops occurs during cell division. During chromatin compaction in the prophase and prometaphase of mitosis, the interphase loops are replaced by a more homogeneous type of loop compaction generated by condensin I and condensin II, loop-extruding complexes that are structurally and mechanistically similar to cohesin [10]. During entry into G1-phase the cohesin-dependent loops are re-established [11] with cohesin-mediated loop extrusion ongoing during the entire interphase. Thus, all types of chromatin loops are dynamic and are re-organized during the cell cycle and by transcriptional cues during differentiation and development.

The loops provide a dynamic structural scaffold to chromatin and impact gene expression, by directly recruiting enhancers to promoters or indirectly through confining the search space of enhancers.

A general depletion of cohesin or CTCF in cells only leads to mild misexpression of a few hundred genes [12], indicating that TAD chromatin loops are only one layer for controlling gene expression. However, disruption or reorganization of specific loops has been shown to impair development in mouse models [13] and to be linked to diseases, including cancer (e.g., enhancer hijacking in AML [14]) and to developmental defects [15].

Chromatin conformation capture methods (such as 4C, Hi-C, Micro-C or GAM) cannot show the dynamics of chromatin loops since they analyze millions of cells simultaneously; while available single cell Hi-C data [16] as well as super-resolution chromatin tracing with DNA-FISH [17, 18] indicate cell-to-cell variability with respect to presence and position of loops. Moreover, all these methods miss out the fourth dimension—time—since they are carried out on fixed cells.

The observation and eventual manipulation of chromatin contacts in live cells by imaging techniques provides a new perspective on chromatin dynamics. Fundamental questions can be approached from a different angle, for example the dynamics of promoter–enhancer interactions, the stability and duration of looping interactions, the role of proteins (in addition to CTCF and cohesin) and protein aggregates/droplets (e.g., transcription factories) for stabilizing or destabilizing specific loops and chromatin-reorganizing processes (replication and cell division). These observations require the labeling of DNA loci with fluorophores and live-cell imaging at high resolution. Major challenges include the selection of suitable labeling approaches to obtain a high signal-to-background ratio that permits visualization and tracking of the signals, to allow imaging over long periods of time (see also the review by the Hansen lab) [19]. In this review, we will discuss the different approaches successfully used to label DNA loci in live cells and highlight novel insights gained with these techniques.

### Single locus labeling approaches

#### Fluorescent repressor operator systems (FROS)

The first approach used to visualize specific sequences in mammalian cell nuclei was the lac operator–repressor system. In a pioneer study, Robinett et al. [20] inserted a mammalian DHFR expression vector with 256 lac repeats randomly into the genome of Chinese hamster ovary cells and demonstrated that the genetically amplified operator array gives a comparable chromatin staining after in situ hybridization, immunostaining with exogenous lac repressor protein after fixation and live cells by expressing the lac repressor protein fused to a fluorescent protein and a nuclear localization signal. Moreover, in the absence of gene amplification but with appropriate nuclear expression levels, a single copy insertion of 256 lac repeats provided sufficient signal-to-background signal to detect the locus as a diffraction limited spot (Table 1) [20].

**Table 1:** Summary of the advantages and disadvantages of the discussed DNA labeling methods

Method and signal amplification method	Advantages	Disadvantages	References
**Fluorescent repressor/operator systems (FROS)** Signal amplification used: SunTag system [91]	· Sufficient signal with insertion of at least 96 operator repeats · Simultaneous labeling of several loci possible by insertion of different arrays	· Requires genome editing · Can lead to genomic instability, heterochromatin formation and gene silencing · An block transcription when placed in an intron · Relatively large arrays (several kbs)	[20–28]
**Transcription activator-like effectors (TALE)** Signal amplification used: quantum dots [34]	· Highly versatile · Simultaneous labeling of several loci possible · No genome editing	· Ideal for repetitive sequences · Design and generation of multiple TALEs for targeting a locus labor intensive · Shown to work for single loci only in combination with quantum dots	[29–35]
**dCas9 coupled to fluorophores (dCas9)** Signal amplification used: RNA aptamer signal amplification [48–51] SunTag system [42–44] Casilio [50, 53] ArrayG [47]	· Highly versatile · Simultaneous labeling of several loci possible · No genome editing	· Low signal-to-background ratio · Can block transcription when placed on the wrong DNA strand	[36–53]
**ANCHOR** Signal amplification used: none described	· Inserted sequence is relatively small · Combination of ANCHOR systems can be used simultaneously · No genomic instability · Can be inserted close to promoters, enhancers and intragenic	· Requires genome editing · Not all ANCHOR systems work equally efficiently	[54–57]

The use of the Lac repressor–operator system was followed by the successful adaption of several other repressor–operator combinations (TetR, λcI, MalI and CymR) [21, 22] which allowed researchers to simultaneously visualize multiple genomic loci at diverse locations in live cells for the first time.

In these initial FROS experiments, repressor proteins were used because their high affinity to the operator binding sites would create a spot with a detectable signal-to-background ratio. However, it was shown later that the tight binding of LacR and TetR molecules to their operator sequences can have undesired biological consequences such as gene silencing. In the human osteosarcoma U2OS cell line, insertions of lac repeats were shown to induce an arrest in early S-phase, possibly by stalling DNA polymerase [23]. The same replication roadblock effect was induced by the introduction of a tet operator array in *Escherichia coli* [24]. In mouse embryonic stem cells, it was reported that the insertion of lac operator arrays next to the D_H_J_H_ elements and the Eμ enhancer of the immunoglobin locus failed to result in germline transmission. The integration of the lac operator array in mouse embryonic stem cells resulted in an abnormal chromosome count of 70–80 chromosomes, indicating that the insertion of the lac operator array can lead to chromosome instability [25].

As shown by these examples, FROS labeling might induce genomic instability, and the effect of genomic insertion of FROS arrays needs to be carefully controlled.

Before the advent of CRISPR-based genome editing, the use of operator–repressor arrays to image specific loci of interest was largely limited to species with high rates of homologous recombination such as *Saccharomyces cerevisiae* [26]. In mammalian cells, FROS arrays were typically randomly inserted into the genome. With the increased possibilities of genome editing by homologous recombination and more recently CRISPR–Cas9 directed insertion of different operator–repressor assays within the same locus or chromosome became a possibility. However, the large size and repetitive nature of the operator arrays (typically 196–256 operator binding sites making up a total of 7–12 kb) still posed an experimental hurdle for facile CRISPR-based genome integration. Alexander et al. circumvented this problem by first integrating two AttP landing sites recognized by the bacteriophage integrases PhiC31 and Bbx1. This was followed by the integration of a plasmid containing the operator array sequence and an antibiotic resistance gene, which was removed later by CRE-lox or FLP-FRT recombination [27]. Tasan et al. developed a method of synthesizing a 96-mer TetO array with varying intermittent spacer sequences. This optimized TetO array of approximately 3 kb length can be inserted via CRISPR–Cas9 in a genomic location of interest and no perturbation of the nuclear localization or chromatin state of the targeted loci was observed. By employing a mutant TetR protein and mutated TetO sequence, even the multiplexing of two TetR type FROS systems in mammalian cells became possible [28].

#### Transcription activator-like effectors (TALEs)

Transcription activator-like effectors (TALEs), discovered in the plant pathogenic bacteria *Xanthomonas*, are proteins that bind to specific DNA sequences [29]. TALEs bind to DNA through a domain of 32 to 35 amino acids called repeat-variable di-residues (RVDs) with two hypervariable amino acids, each of them binding to a specific base pair. Through DNA cloning, the amino acid code can be reprogrammed to bind specific DNA sequences. This versatility made TALEs a popular platform for applications such as gene editing and transcriptional modulation [30, 31].

Since TALEs can be modified to bind specific DNA sequences, it has also been an attractive method to fluorescently label such sequences (Table 1). Several studies have successfully labeled repetitive sequences like telomeres and centromeres in different circumstances. For example, Miyanari et al. demonstrated that TALEs can be used to study chromatin dynamics throughout the cell cycle [32]. Ren et al. have studied age-associated genomic alterations at telomeres and centromeres in premature aging models in both human and mouse. Others have labeled major satellite repeat regions and followed the dynamics of chromosomes through the cell cycle or were able to differentially label both parental chromosomes in hybrid mouse cells [31, 33].

While studies on repetitive sequences have provided valuable insights, it is still limited to a set of specific repetitive regions. To research TAD boundaries or enhancer–promoter interactions, it would be necessary to label non-repetitive single loci. Ma et al. demonstrated that TALEs can be used to visualize single DNA loci by mapping HIV-1 proviral DNA sequences in the human genome using TALEs labeled with quantum dots (QDs), which have unique optical properties including excellent brightness and photostability compared with traditional organic dyes [34]. The QDs greatly improve the signal-to-background ratio, reducing the number of fluorescent particles necessary to be detectable. However, a disadvantage is that TALE-QD particles need to be delivered to cells by transfection for each experiment, which limits their application.

While the other methods mentioned in this review are currently more prevalent, TALEs can be a valuable tool for DNA visualization. It might be interesting to develop a repetitive array, similar to FROS arrays, that can recruit a set of the same TALEs without the need to develop many different TALEs each targeting a specific DNA sequence. This would yield another system that can be used in combination with other DNA labeling systems such as FROS, dCas9 or ANCHOR. In addition, in cases were the binding of repressors to operator arrays induces replication roadblock effects, TetO targeting TALEs could be an alternative. An advantage of TALEs is that the residence time of TALEs can be adjusted by varying the length of the DNA binding domain, whereas repressors can currently only be altered by previously described mutations [35].

#### CRISPR/deadCas9 (dCas9)

**Fig. 2:**
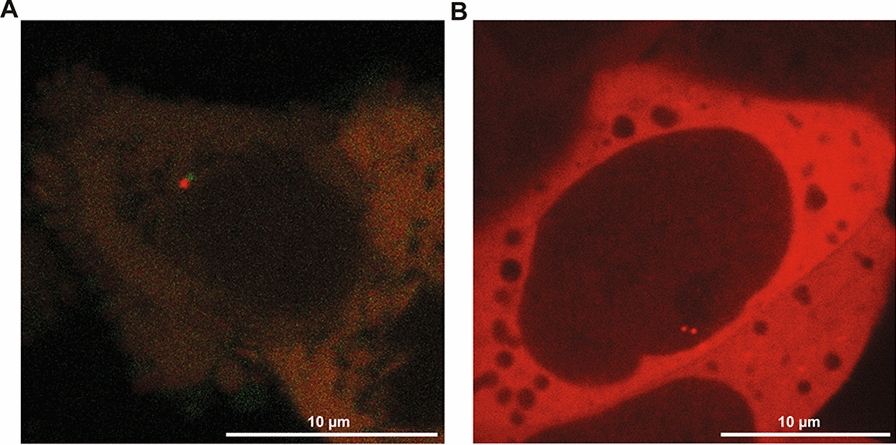
Example image of ANCHOR and dCas9 labeling **A**: Image of a live HCT116 cell with two labeled loci, the D4Z4 repeats and a neighboring TAD boundary that are roughly 15 Mbp apart on chromosome X. The D4Z4 repeats were labeled with and a repeat-specific gRNA and dCas9-eGFP(green), the neighboring TAD boundary was visualized with an ANCH3 insertion in combination with the OR3-HaloTag protein and JF-549 dye (red). The image was taken on a SP5 Leica confocal microscope. **B**: Image of a live mouse embryonic stem cell with a locus on chromosome 4 visualized by an ANCH3 insertion and OR3-Halo/JF-646. The image was taken on a SpinSR SoRa Olympus spinning disk microscope. Note that the locus was already replicated and the two sister chromatids are visible

The CRISPR/Cas9 (Cas9 for short) genome editing tool that revolutionized biomedical research can be repurposed for labeling of genomic regions. Originally an antiviral defense system of prokaryotes, the Cas9 protein together with a guide RNA (sgRNA) sequence can induce a double stranded break with near base pair precision [36]. This made Cas9 the gold standard of genome editing [37]. Catalytic inactive mutants of Cas9 (deadCas9 or dCas9) turned out to be also quite versatile tools [38]. Since the dCas9 cannot cleave the target DNA, it is not released from chromatin. This long residence time makes it a very attractive tool to recruit proteins or protein domains, such as fluorescent tags or gene silencing domains, to specific genomic loci (Fig. 2). A clear advantage of dCas9-mediated labeling over other methods covered in this review (see Table 1) is that genome editing is in general not necessary, making it relatively straightforward to use on many different loci as well as in cell lines that are difficult to edit.

Chen et al. were the first to describe imaging of telomeres with dCas9-eGFP in combination with one sgRNA [39]. They also labeled the non-repetitive regions of the *Muc4* gene with combinations of 26 or more sgRNAs. A similar approach was used to label enhancers and promoters with 36 sgRNAs each [40]. An alternative approach involving genome editing is the CRISPR-tag, developed by Chen and colleagues [41]. They inserted a repeat of short CRISPR target sites derived from the *Caenorhabditis elegans* genome (up to 6 repeats with at least 24 sgRNA sites) in the genomic region of interest [41] to recruit sufficient dCas9 molecules.

While it is very attractive to use dCas9 to label a specific locus, a major problem of dCas9-FP-based methods remains the low signal due to the limited number of fluorophores that can be recruited to the locus. Some groups succeeded in amplifying the signal by fusing the supernova tagging system (SunTag) to dCas9 [42–44]. The SunTag is a peptide scaffold with multiple GCN4 epitopes (e.g., 24) that can be bound by a single-chain variable fragment of an anti-GCN4 antibody. The nanobody can be tagged with fluorophores and expressed in mammalian cells (scFv-GCN4-GFP). When the SunTag system is fused to dCas9, this enables a substantial amplification of the fluorescent signal. This technique has been shown to work with guideRNAs for repetitive sequences as well as on non-repetitive regions such as the *MUC4* gene [39, 45–47].

Alternative approaches that allow the recruitment of a large number of fluorophores per dCas9 molecule involve extending the sgRNA with RNA aptamer repeats such as repeats of the MS2 RNA binding sequence, bound by fluorophore-fusions of the MS2 bacteriophage coat protein, or other analogues [48–51]. Each RNA aptamer recruits two fluorescent proteins, leading to an accumulation of fluorescent signal at the target sequence. Combining different RNA aptamer analogues enables multi-color imaging. Maass et al. successfully labeled two different alleles in 129S1/CAST hybrid mouse embryonic stem cells (mESCs) and mouse embryonic fibroblasts (MEFs) by using single nucleotide polymorphisms in PAM sites [52]. This allowed them to confirm that single loci as well as chromosomes are stably positioned in space and time. Kamiyama et al. developed a splitGFP-system consisting of strands 1–10 of the GFP beta-barrel structure that only becomes fluorescent when it is complemented by the 11th beta-strand, which can be fused as a tagging sequence to a protein of interest [45]. Ghosh et al. [46] developed the GFP enhancer nanobody array (ArrayG) that can be attached to dCas9. Wild-type monomeric GFP molecules are initially dim but increase about 26 times in brightness when bound to the nanobody array. This system was used to track a repetitive genomic locus in live cells [47]. Clow et al. successfully adapted a dCas9-based platform that they previously used to label repetitive sequences (Casilio) [50, 53]. This system uses PumilioFBF (PUF)-tethered factors which can be engineered to bind to a specific 8-mer at the 3’end of a gRNA. By multiplexing the 8-mer sequences, PUF domains fused with fluorescent domains can accumulate at the dCas9. With this approach, they successfully labeled three different non-repetitive loci with three different colors. They demonstrate the applicability of Casilio for chromatin interaction studies by labeling the *IER5L* promoter and its super-enhancer, a known ~ 500-kb chromatin interaction dependent on RAD21 [5]. After degradation of RAD21, they indeed detected an increased distance between the labeled loci. The adaptations mentioned above use fluorophores (antibody-FP, MCP-FP and PUF-FP) which stochastically bind to the dCas9 fusion protein. This renders these systems inherently more resistant to photobleaching than dCas9-FP labeling schemes, paving the way for acquiring longer time tracks.

The advances on the dCas9 labeling techniques have significantly improved its signal-to-background ratio, resistance to photobleaching and ability to visualize loci over long time periods. Successful dCas9 labeling in these systems requires the expression of multiple genetic components (dCas9, binder and sgRNA(s)) either via transient transfection or the generation of stable cell lines. While dCas9 imaging systems can be relatively easily established on telomeres or other repetitive genomic loci, the step to label a single, non-repetitive locus is often large. This can be explained by the delicate balance of getting both a sufficient fluorescent protein level to label the locus and avoiding a high background signal that could obstruct the single locus signal. Expressing the fluorescent moiety at a low but sufficient level is therefore almost always an advantage in obtaining a high signal-to-background ratio, because expressing the fluorescent molecule at high levels increases the number of molecules in the background while the number of locus-binding molecules remains the same (small) number, certainly when single non-repetitive loci are targeted. At best the binding equilibrium at the target is somewhat improved, but this will be negligible relative to the increase in background fluorescent molecules, particularly when the fluorescent molecule has a long residence time at the target sequence. The flexibility of the dCas9 system has a great appeal for single locus tracking techniques, and ultimately a combination of solutions as described above might improve robust labeling of single loci.

#### The ANCHOR DNA labeling system

In 2014, Saad et al. introduced a novel protein–DNA imaging approach called the ANCHOR DNA labeling system [54]. This labeling approach leverages the ParB–ParS binding of the ParABS partitioning system naturally occurring in bacteria. In the ANCHOR DNA labeling approach, fluorescently labeled ParB molecules (ORs) recognize a small set of ParS sites within a short non-repetitive INT sequence (ANCH) which can be integrated next to the locus of interest. Upon recognition of the ParS site by a ParB homodimer, a conformational change of the ParB molecules leads to N-terminal mediated oligomerization and spreading of ParB along the adjacent DNA strand via non-specific interactions. Together, the binding and spreading of fluorescent ParB proteins creates a diffraction limited spot at the locus of interest (Fig. 2) [55, 56]. A key advantage of the ANCHOR DNA labeling system is that it has been reported to not inhibit transcription even when present directly adjacent to a promoter or an intronic enhancer [55, 57].

Since different bacteria strains and replicons (e.g., genomes and plasmids) within the same bacterium contain different ParB-INT combinations, multiplexing non-crossreactive ANCHORs to label multiple loci became possible. For example, the ANCHOR1 and ANCHOR2 system were used in combination to visualize the dynamics of double strand break repair in *Saccharomyces cerevisiae*. Later, the ANCHOR1–ANCHOR3 pair was used in mouse embryonic stem cells to investigate the heterogeneity of chromatin dynamics [58]. Recently, significant differences between the labeling efficiency of different ANCHORs have been reported. A survey of single locus tracking techniques in *Drosophila* showed that ANCHOR2 labeled two regions of interest (ROIs) equally efficiently as LacI-LacO labeling, whereas ANCHOR1 showed less efficient labeling at both ROIs. In addition, both ANCHOR systems showed sensitivity to the design of the ParB-FP construct and cellular context [57]. This suggests that the extent of binding and/or cis-spreading of different ANCHORs might be tissue-, cell- and locus-dependent.

The ANCHOR DNA labeling system shows great promise as the latest addition to the available single locus tracking techniques. The ease of CRISPR-based genomic insertion of the short ANCH sequences (~ 1 kb) in combination with the ability to non-invasively label genomic regions of interest make the ANCHOR DNA labeling system a promising tool for studying locus dynamics (see Table 1). In the future, optimizations to the ParB-FP fusion design might further improve the labeling efficiency and robustness of the existing ANCHOR systems and novel ANCHOR types and combinations may be developed.

## Insights into the dynamics of higher order chromatin structures using single locus labeling approaches

Several groups have demonstrated that the single locus labeling approaches described above are very powerful to study the dynamics of chromatin interactions, including also promoter–enhancer interactions.

Gabriele et al. studied a 505 kb TAD containing only the *Fbn2* gene, which is inactive in mESCs. They used a combination of ANCH3 and TetO array integrations to achieve dual color labeling [59]. Interestingly, they found that the *Fbn2* loop is rare and highly dynamic. The fully looped state was present in only ~ 3–6,5% of the analyzed cells and the median loop lifetime of the *Fbn2* loop is 10 to 30 min. Given that the *Fbn2* structural loop appears as a relatively strong interaction in Hi-C, suggesting a frequent occurrence of the loop, this unexpected result shows that live-cell imaging of genomic loci gives a very different perspective on the interpretation of Hi-C results.

Mach et al. used live-cell imaging to specifically study the effect of CTCF and cohesin on TAD dynamics [60]. They introduced LacO and TetO arrays containing removable 3xCTCF sites separated by 150 kb in a TAD (560 kb) without active or inactive genes. In presence of the 3xCTCF sites and RAD21, they found that the arrays spent 78% of the time in close contact, while this was reduced to 33% when the 3xCTCF sites were removed and 23% after RAD21 depletion. The average contact time and formation rate decreased from a contact lasting 16 min and reforming every 5 min to an average contact duration of 6 min reoccurring every 10 min after removal of CTCF sites, and 2 min encounters with an average reformation rate of 22 min upon RAD21 depletion. Thus, CTCF and cohesin greatly impact the stability and frequency of contacts with a genomic separation distance of 150 kb and 500 kb [59]. Both studies suggest that CTCF–CTCF contacts are transient and that CTCF-dependent TAD structures in single cells are highly dynamic entities.

Active transcription has been widely associated with 3D enhancer–promoter proximity. Using a dual locus labeling of the *Sox2* promoter and *Sox2* control region in mouse embryonic stem cells with the tetO/TetR system as well as the novel cuO/CymR pair (repressor system from *Pseudomonas putida*), Alexander et al. have excluded the presence of a long-lived state in which the enhancer and promoter physically interact [27]. Similar observations have been reported by Platania et al. for the same locus using ANCHOR1/3 dual color labeling [61]. This is actually in line with earlier observations at the ß-globin locus in which the locus control region can dynamically flip-flop between downstream promoters at a time scale of minutes [62]. We anticipate that future studies will quantify the spatiotemporal dynamics of enhancer–promoter pairs similar to recently published studies on the dynamics of architectural loops [59, 60].

Several studies show an interplay between transcription activity and locus mobility. For example, Gu et al. have shown with a dCas9-based labeling approach that enhancers and promoters diffuse at a faster rate when a locus is transcriptionally active in comparison to an inactive locus [40]. Using a combination of ANCHOR3 labeling at the promoter and visualization of nascent transcripts with MS2 repeats, Germier et al. showed that transcriptional activation of a transgene leads to the confinement of its promoter [55]. At the *Sox2* locus, not only the promoter and the *Sox2* control region (SCR) displayed confined motion, also the intermittent and downstream genomic region showed a higher level of confinement [61] than a transcriptionally inactive region (*HoxA* locus) measured in the same cell type [58].

The abovementioned results indicate that transcriptional activation of a locus can have effects on the mobility of regulatory and non-regulatory elements. However, nascent transcription has been shown to occur in transcriptional bursts [62]. Studies which simultaneously visualize mRNA and chromatin in live cells show that whereas for some loci the bursting gene is on average more confined than the non-bursting gene, no differences in mobility were observed for other loci [63, 64]. Similarly, it was shown that the *Sox2* enhancer diffuses more freely at transcriptionally poised alleles compared to transcribing alleles, whereas the level of confinement of the *Sox2* promoter remains similar [61].

In the future, experiments in which the movement of single loci are tracked across a longer time window spanning multiple transcriptional events might increase our understanding of the relationship between active transcription and chromatin dynamics.

### Selection and optimization of single locus labeling approaches

As the insertion of a fluorogenic array or the fluorescent labeling of an endogenous genomic region has the potential to disrupt the genomic feature of interest, the choice of the place for insertion and labeling a region is a careful balancing act between minimizing the linkage error without causing perturbation. In a recent article, Brandão et al. simulated the effect of label placement and localization error on the ability to detect a 500 kb region in a looped and nonlooped state. When the linkage and localization error become too large, looped and nonlooped states can no longer be detected by locus tracking experiments [19].

In endogenous contexts, loop anchors such as enhancers, promoters and CTCF sites have been successfully tracked by FROS, dCas9-based and ANCHOR approaches with linkage distances ranging from several kilobases to 10 kb [27, 40, 57, 59, 65].

Another important consideration for selecting the type and the position of the label is the impact on the chromatin context for gene regulatory elements and gene expression. For example, Delker et al. found that in *Drosophila* cells, the presence of LacI molecules at a 20mer LacO array placed at the *Ubx* intronic enhancer decreased Ubx protein levels, whereas no perturbation was observed when using the ANCHOR1 and ANCHOR2 labeling technique [57]. In line with this observation, strong DNA binding of wild-type TetR to a 96 × TetO repeat array reduced transcription when it was inserted into an intron of the *Nanog* gene as part of the STREAMING tag system [64]. Therefore, arrays involving strong DNA binding proteins have disadvantages when placed within genes.

Approaches based on dCas9 can avoid interference with ongoing transcription by targeting the template strand, since guide RNAs targeting the non-template strand have a higher gene silencing effect compared to those targeting the template strand [64, 66].

Important limitations of all approaches are the signal-to-background ratio as discussed above and the stability of the selected fluorophores against fluorescence bleaching. Depending on the imaging setup, these factors limit the experiment and complicate image analysis. One system that efficiently increases the signal-to-background ratio are fluorogenic tags, green fluorescent protein–nanobody arrays, and monomeric wild-type green fluorescent protein binders that are initially dim but brighten ~ 26-fold on binding with the array [46, 47] (see also Lu et al. [67] for an in-depth discussion of signal amplification methods). The advantage of such multicomponent approach is also that bleached fluorophores can be replenished at the target site over time.

## Perspective

The single locus labeling approaches covered here will contribute to solving major questions around the dynamics of the chromatin fiber. Success depends on the right choice of the labels (reviewed here, see also [68]) and equally on the available imaging acquisition setup and the image analysis tools (reviewed in [19]). The ongoing continuous improvement of imaging technology in combination with the improvement of the fluorescent labels and protein manipulation tools (e.g., rapid protein depletion systems and optogenetics) will overcome some of the limitations faced by the current studies, making currently impossible experiments feasible in the near future.

Looking ahead, one important problem is the correlation of enhancer–promoter proximity with transcription, which was suggested in many biological systems [69–73] but is mechanistically not understood yet. The ability to visualize an enhancer, promoter and nascent RNA in single live cells has led to the notion that live-cell imaging approaches could be used to test models of enhancer–promoter communication. In *Drosophila*, transcriptional activation by a distal enhancer only occurred when the enhancer and promoter were in a proximal state (< 340 nm) [74]. In contrast, in mouse embryonic stem cells, tracking of the *Sox2* enhancer control region (SCR), promoter and nascent RNA revealed no correlation between enhancer–promoter proximity and nascent transcription. This study of Alexander et al. revealed no evidence of sustained physical contact between enhancers and promoters. The authors propose that the absence of a correlation between enhancer–promoter proximity and *Sox2* transcription might be the result of long-lived activation of the promoter, long-range gene activation, for example by formation of large transcription factor condensates, or by limitations in the spatiotemporal resolution of the experiment [27]. We anticipate that continuous improvements on the precision of single locus tracking techniques by (fluorogenic) signal amplification strategies and the increase in length and temporal resolution of dual locus tracks obtained by light sheet imaging and point spread function (PSF) engineering ([47], reviewed in [75]) will lead to the first estimates on the frequency and duration of enhancer–promoter contacts in mammalian cells. A number of observations reinforce the importance of contextual chromatin contacts on enhancer–promoter interactions and transcription. At the *SOX9* locus, interactions between structural elements positioned between the enhancer cluster and the promoter facilitate enhancer–promoter communication and transcription [76]. At the mouse α-globin cluster, deletion of CTCF binding sites led to ectopic contacts and aberrant activation of neighboring genes by α-globin enhancers [77]. Similarly, in some chromatin contexts promoters seem to compete for the same enhancer [62], whereas at other loci multiple promoters co-assemble in regulatory hubs [78].

Revisiting these observations by investigating chromatin contacts in live single cells and eventually the combination of DNA, RNA and protein labeling to visualize all components contributing to the nanoscale organization of the cell nucleus, will lead to a deeper understanding of gene regulatory mechanisms.

Many proteins have been shown to accumulate in transient (seconds) and stable foci (minutes to hours) in which the individual molecules exchange within seconds [79–82]. Dual imaging of foci and single molecules enables the detection of mobility patterns of these molecules inside and outside these foci [83], but much more information can be gained when single particle tracking is combined with the genomic context (e.g., promoters, enhancers) by labeling of genomic loci. The latter experimental setup opens the possibility of determining the biophysical properties of proteins visiting individual genomic loci, for example during a target search [84].

In addition, recent advances in super-resolution techniques such as STED have enabled the detection of sub-diffraction sized clusters of regulatory factors proximal to transcription sites [64, 65, 73, 85]. Manipulation of biomolecules by mutations, their rapid nuclear depletion and/or small molecules are used to reveal which players and protein domains are essential for foci formation and stabilization [65, 86–88].

In the future, similar perturbation experiments can be performed to evaluate foci formation and stabilization at specific genomic locations. Alterations, deletions, inversions and rearrangements of DNA sequences powered by novel synthetic regulatory genomic approaches [89] combined with super-resolution imaging of foci and single loci might provide insights in the role of individual regulatory factors and enhancer elements in the formation of foci at enhancer clusters [90].

Ultimately, these experiments might not only reveal to what extent foci influence the biophysical behavior of individual molecules, but also how foci and the number of individual molecules within foci are related to the output of local molecular processes such as DNA damage repair, DNA replication and transcription.

## Data Availability

This is a review article, there are no data linked to the manuscript that can be made publically available.
